# Estimating the household secondary attack rate and serial interval of COVID-19 using social media

**DOI:** 10.1038/s41746-024-01160-2

**Published:** 2024-07-20

**Authors:** Aarzoo Dhiman, Elad Yom-Tov, Lorenzo Pellis, Michael Edelstein, Richard Pebody, Andrew Hayward, Thomas House, Thomas Finnie, David Guzman, Vasileios Lampos, Virus Watch Consortium, Rob Aldridge, Sarah Beale, Thomas Byrne, Jana Kovar, Isobel Braithwaite, Ellen Fragaszy, Wing Lam Erica Fong, Cyril Geismar, Susan Hoskins, Annalan Navaratnam, Vincent Nguyen, Parth Patel, Maddie Shrotri, Alexei Yavlinsky, Pia Hardelid, Linda Wijlaars, Eleni Nastouli, Moira Spyer, Anna Aryee, Rachel McKendry, Tao Cheng, Anne Johnson, Susan Michie, Jo Gibbs, Richard Gilson, Alison Rodger, Ingemar J. Cox

**Affiliations:** 1 Department of Computer Science, University College London, London, UK; 2 Centre of Excellence for Data Science, AI and Modelling, University of Hull, Hull, UK; 3 Microsoft Research, Herzliya, Israel; 4 Department of Computer Science, Bar Ilan University, Ramat Gan, Israel; 5 Department of Mathematics, University of Manchester, Manchester, UK; 6 Azrieli Faculty of Medicine, Bar-Ilan University, Safed, Israel; 7 UK Health Security Agency, 61 Collingdate Avenue, NW9 5EQ London, UK; 8 UCL Collaborative Centre for Inclusion Health, UCL, London, UK; 9 Department of Computer Science, University of Copenhagen, Copenhagen, Denmark; 10 UCL Institute Of Health Informatics, University College London, London, UK; 11 UCL Institute of Epidemiology and Health Care, University College London, London, UK; 12 Extreme Events and Health Protection Team, Centre for Radiation, Chemicals and Environmental Hazards, Public Health England, London, UK; 13 Department of Infectious Disease Epidemiology, London School of Hygiene and Tropical Medicine, London, UK; 14 MRC Centre for Global Infectious Disease Analysis, Department of Infectious Disease Epidemiology, School of Public Health, Imperial College London, London, UK; 15 Department of Population, Policy and Practice, UCL Great Ormond Street Institute of Child Health, University College London, London, UK; 16 Francis Crick Institute, London, UK; 17 University College London Hospital, London, UK; 18 London Centre for Nanotechnology and Division of Medicine, University College London, London, UK; 19 Department of Civil, Environmental and Geomatic Engineering, University College London, London, UK; 20 Centre for Population Research in Sexual Health and HIV, Institute for Global Health, London, UK; 21 Centre for Behaviour Change, University College London, London, UK; 22 Institute for Global Health, University College London, London, UK; 23 Royal Free London NHS Foundation Trust, London, UK

**Keywords:** Viral infection, Risk factors

## Abstract

We propose a method to estimate the household secondary attack rate (hSAR) of COVID-19 in the United Kingdom based on activity on the social media platform X, formerly known as Twitter. Conventional methods of hSAR estimation are resource intensive, requiring regular contact tracing of COVID-19 cases. Our proposed framework provides a complementary method that does not rely on conventional contact tracing or laboratory involvement, including the collection, processing, and analysis of biological samples. We use a text classifier to identify reports of people tweeting about themselves and/or members of their household having COVID-19 infections. A probabilistic analysis is then performed to estimate the hSAR based on the number of self *or* household, and self *and* household tweets of COVID-19 infection. The analysis includes adjustments for a reluctance of Twitter users to tweet about household members, and the possibility that the secondary infection was not acquired within the household. Experimental results for the UK, both monthly and weekly, are reported for the period from January 2020 to February 2022. Our results agree with previously reported hSAR estimates, varying with the primary variants of concern, e.g. delta and omicron. The serial interval (SI) is based on the time between the two tweets that indicate a primary and secondary infection. Experimental results, though larger than the consensus, are qualitatively similar. The estimation of hSAR and SI using social media data constitutes a new tool that may help in characterizing, forecasting and managing outbreaks and pandemics in a faster, affordable, and more efficient manner.

## Introduction

The household secondary attack rate (hSAR) of a disease measures its potential for spread in the context of repeated close contacts as seen in households, in contrast to measures of overall infectiousness such as the basic reproduction number *R*_0_. The hSAR can be defined in different ways, but here we use the common definition as the probability of a household member acquiring the disease within an incubation period given another household member is infected. The investigation of hSAR, in addition to reproduction rate, is important to understanding the risk of transmission^1^, and also to inform interventions such as the decision to vaccinate close contacts of immunocompromised individuals^2^. The household SAR is affected by a number of parameters including the disease (e.g. its reproduction number, incubation period, variants of concern)^3^, the number of household members^4^, the size of the dwelling, contact environment^5^, preventive measures of household members (e.g. vaccination, masks, social distancing)^6^, comorbidities of the contacts^7^, and demographic features of the population such as age structure, sex ratio, and ethnicity^8^. Accurately estimating the SAR of a disease is difficult. The conventional epidemiological methodology requires a repeated cycle of case investigation and contact tracing^9^ for a sample population. Infection is usually determined through laboratory-confirmed and self-reported cases.

The value of digital footprints, i.e. data people knowingly or unknowingly generate when using electronic services, to infer information about the health of populations or individuals is now well established. This is the basis for digital epidemiology, i.e. epidemiology that uses data generated outside the public health system and not primarily generated for health purposes. There are a wide variety of digital footprints including social media posts, microblogging (X, formerly known as Twitter (For reasons of clarity, we refer to X as Twitter for the remainder of this paper.)), Web search data, and over-the-counter (OTC) sales of medicines. The use of digital trails for syndromic surveillance dates back to at least 1977 when Welliver et al.^10^ demonstrated a strong correlation between sales of OTC remedies and influenza-like illness rates. Interest in syndromic surveillance increased in the early 2000’s with the US Defence Advanced Research Projects Agency initiative called ENCOMPASS (ENhanced COnsequence Management Planning And Support System) to improve early warning systems to protect against bioterrorism. Contemporaneously, a number of papers demonstrated the utility of Web search data for estimating influenza prevalence^11–14^. Subsequently, various researchers showed that Twitter data could also be used for the same purpose^15–18^. The use of digital trails has not been limited to disease prevalence. Other research has shown its utility for a variety of tasks including identifying adverse drug reactions^19,20^, automatically identifying individuals at higher risk of health events^21^, performing disease diagnosis^22,23^, health behaviour analysis e.g., addictions^24^, and mental health prediction^25^.

The use of Twitter data to infer the hSAR and serial interval (SI) of influenza was described in^26^. At the risk of over-simplifying, the fundamental idea is to identify tweets indicating that a user and/or a household member have influenza. When users tweet first about both themselves and then about a household member having influenza, or vice versa, within a predetermine interval, it is assumed that this is an instance of household secondary infection. The hSAR is then estimated as the ratio of the number of such pairs to the total number of users tweeting that they or a household member has influenza. The SI is determined by the time between the two tweets that indicate a primary and secondary infection. Our work significantly extends this approach through demonstrating its utility to COVID-19, and extending the analytical framework. We created a labelled set of tweets indicating whether a tweet was about a user having COVID-19, a household member having COVID-19, or simply about a household member. This data was then used to train three corresponding classifiers. A probabilistic approach is then used to estimate hSAR for each month or week. Monthly and weekly hSAR for COVID-19 are estimated for the UK using Twitter data from January 2020 to February 2022. Not all secondary infections originate from within the household, and, as the prevalence of an infectious disease increases, the probability of acquiring the disease from outside of the household increases. We therefore introduce an adjustment factor to partially correct for secondary infections originating from outside of the household. It has been previously noted^26^ that Twitter users may have varying reluctance to tweet about a household member. This reluctance can lead to under-reporting of household infection and consequently lower our hSAR estimates. We confirmed that this reluctance exists and adjusted for variations in the likelihood of tweeting about a household member. A further contribution is an analysis of the sensitivity to variations in the size of the monthly cohort, and providing weekly as well as monthly hSAR estimates.

Our methodology assumes all households are of size two, based on the UK average of 2.3, and that a Twitter user is only infected once. The rationale and consequences of these assumptions are described in the Discussion.

## Results

### Twitter cohort classification

We first identified a seed group of 1,226,509 Twitter users that issued experiential tweets that included statements of the form “I have COVID” or “My spouse has COVID”. We then performed geofiltering, described in Section 4.1 to only include users based in the UK, resulting in 77,016 users.

Three classifiers were trained to independently identify whether a tweet was (i) about the user/Tweeter having COVID-19, (ii) about a household member having COVID-19, or (iii) about a household member (irrespective of topic), see Supplementary material (Supplementary Methods S 5) for details. The classifiers, denoted C1, C2, and C3, respectively, were trained on a labelled set of 7894 tweets and achieved AUC scores of 0.907, 0.934, and 0.768, respectively. The F1 scores for the three classifiers were 0.8538, 0.8733, and 0.7202 respectively. We denote the probability of tweeting about a household member as *p*_*h*_. The *p*_*h*_ for each user is estimated as the number of tweets classified as positive by C3, i.e. a tweet about a household member, irrespective of topic, divided by the total number of tweets downloaded for this user. We removed users who never tweeted about a household member, i.e. none of a user’s tweets was positively classified by C3, reducing the total number of users (cohort size) to *n* = 58, 555. We report results for *p*_*h*_ > 0 unless otherwise stated. The most recent (up to) 3200 Tweets of these 58,555 users were downloaded. This resulted in 107,921,029 tweets.

**Fig. 1:**
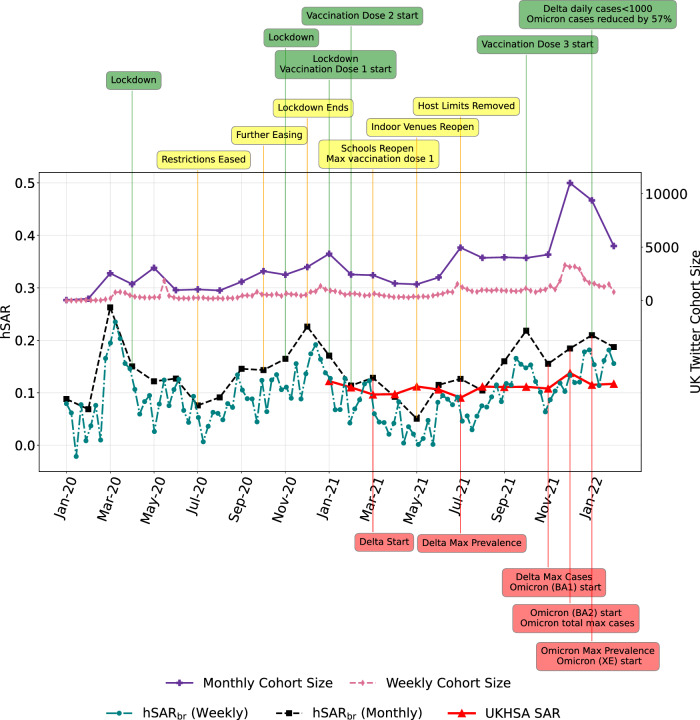
Monthly/weekly household secondary attack rates with key events for the period from January 2020 to February 2022. Monthly and weekly cohort sizes and household SAR (hSAR_br_) marked with key events for the period from January 2020 to February 2022, inclusive, for users with *p*_*h*_ > 0 and an assumed maximum serial interval of 14 days. The UK Health Security Agency (UKHSA) (formerly Public Health England) SAR scores are the weighted average of SAR scores for different variants published by UKHSA.

For each monthly or weekly period, we then identified the subset of users with one or more tweets originating in this time interval and being positively classified by C1 and/or C2, i.e. who tweeted that they and/or a household member had COVID-19. Figure 1 depicts the monthly and weekly cohort size for the period from January 2020 to February 2022, inclusive.

### Household secondary attack rate estimation

Based on this cohort, Fig. 1 also shows the inferred monthly and weekly hSAR estimates (hSAR_br_), where it is assumed that household secondary infection occurs within 14 days of the primary infection. This period is the assumed maximum serial interval. Experiments with a longer assumed serial interval produced very similar results. The time of onset of the primary and secondary infections is assumed to be the dates of the corresponding tweets. UKHSA estimates for hSAR for the period from January 2021 to February 2022 are also reported for comparison purposes. Note that UKHSA did not report hSAR estimates prior to January 2021. See Supplementary Methods S 3 for further details.

### Sensitivity analysis

**Fig. 2:**
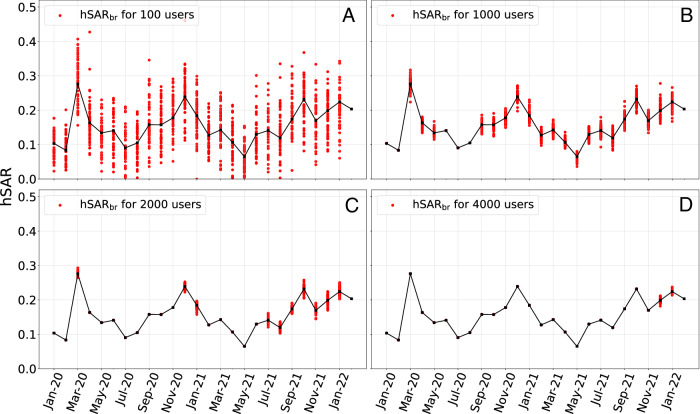
Comparison of hSAR_br_ across for various cohort sizes. The solid line depicts hSAR_br_ values calculated using all the users in our Twitter cohort. The dotted values show hSAR_br_ for fixed cohort sizes of **A**. 100, **B**. 1000, **C**. 2000 **D**.4000 users. For a given cohort size, we create 50 cohorts, uniformly sampled from the available data, total cohort size permitting.

Figure 2 examines the sensitivity of the hSAR_br_ estimates to the cohort size. We note that for the period from January 2020 to February 2022 the three smallest monthly cohort sizes were 48 (January 2020), 173 (February 2020), and 947 (August 2020). The average (median) monthly cohort size was 3143.19 (2497).

### Adjustment for infection outside of the household

**Fig. 3:**
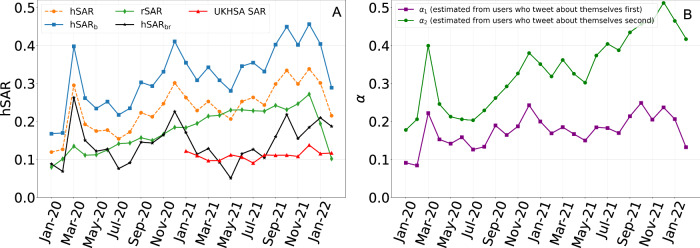
Adjustments to the household secondary attack rate estimates. **A** hSAR depicts the monthly household SAR assuming no reluctance to tweet about a household member. hSAR_b_ depicts the household SAR adjusted for reluctance. rSAR is an estimate of second infections from outside of the household. Our final estimate of household SAR is hSAR_br_ = hSAR_b_ − rSAR. **B** The monthly values for *α*_1_ (the hSAR estimate calculated from the subset of users who tweeted about being infected and subsequently tweeted that a household member was infected) and *α*_2_ (the estimate calculated from the subset of users who tweeted about a household member being infected and subsequently tweeted that they were infected) for users with *p*_*h*_ > 0 assuming a maximum serial interval of 14 days.

Our estimate, hSAR_br_, incorporates two adjustments. The first adjustment attempts to account for the probability that the source of the secondary infection was outside of the household. This is of particular concern as our cohort size is determined by the number of index cases tweeting about COVID-19. As such, it is strongly related to the community incidence of COVID-19. When the community incidence is high it is more likely that a second case in the household will in fact have been acquired outside the household, leading to higher levels of estimated hSAR when the cohort size is large. The correlation between our hSAR estimates, denoted as hSAR_br_, and the corresponding monthly and weekly cohort sizes are 0.504 (*p* = 0.0085) and 0.389 (*p* = 2.01e–05), respectively. For comparison, UKHSA SAR estimates have a correlation of 0.5304 (*p* = 0.0509) with their corresponding cohort sizes. Figure 3A illustrates the secondary attack rate, hSAR_b_, before the removal of the probability of non-household secondary infection, the latter probability denoted as *rSAR*. Without this adjustment, the correlation between the estimated secondary attack rate, hSAR_b_, and the corresponding cohort size is significantly stronger at 0.7220.

### Adjustment for reluctance to tweet about household members

The second adjustment attempts to correct for people’s reluctance to tweet about a household member. Previous work^26^ has noted that the probability of tweeting about a household member having influenza-like illness (ILI) *after* tweeting that the Twitter user had ILI, is likely to be less than the converse, i.e. the probability of tweeting that the Twitter user has ILI *after* tweeting that a household member is infected. The former probability is expected to be less due to the reluctance to tweet about a household member. To examine this we computed the hSAR independently for the two groups, as depicted in Fig. 3B. The two curves, denoted *α*_1_ and *α*_2_, represent the hSAR estimates from (1) the users that first tweeted about being infected and then tweeted about a household member being infected, and (2) users that first tweeted about household members being infected and then tweeted about themselves being infected. If there was no reluctance to tweet about household members, we would expect the two curves to be very similar. Instead *α*_1_ is consistently less than *α*_2_, indicating that household infections are being under-reported. Figure 3A depicts the hSAR estimates before and after adjustment i.e. hSAR and hSAR_b_. A plot of *α*_1_ against *α*_2_ (see Supplementary Fig. 1 of the supplementary material) gives a best fit line with gradient 0.56 indicating that household infection is under-reported by about 44%.

### Serial interval estimation

**Fig. 4:**
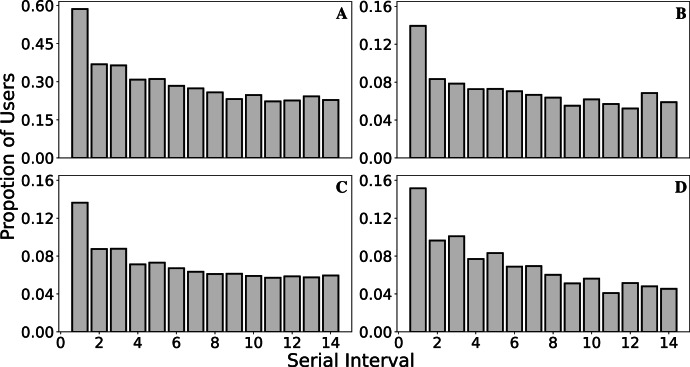
Serial interval for Alpha, Delta, and Omicron dominant periods. Histogram of serial interval for daily bins with an assumed maximum serial interval of 14 days for the periods **A** All the months from Jan 2020 to Feb 2022. **B** Alpha dominant period (18 December 2020 to 15 May 2021). **C** Delta dominant period (22 May 2021 to 19 December 2021). **D** Omicron dominant period (23 December 2021 to 28 February 2022).

Finally, Fig. 4 depicts the distribution of the serial (time) interval between the primary and secondary reports of household infection, aggregated over the entire period from Jan 2020 to Feb 2022 and the periods where the Alpha, Delta, and Omicron variants were dominant. The mean (median) serial intervals are 6.49 (6), 6.67 (6), 6.61 (6), and 6.10 (5), respectively.

## Discussion

Figure 1 shows the monthly/weekly cohort size gradually increasing over time, with a significant jump in November and December of 2021. This increase might be partially attributable to an increase in willingness to publicly reveal/discuss personal and household COVID-19 status. However, we hypothesise that the increase is primarily driven by increases in the incidence of COVID-19 during this period. Based on UK government statistics^27^, the number of reported cases on 2nd November 2021 was 31,328, on 1st December 2021 it was 47,263, and on 1st January 2022 it was 99,304.

As expected, Fig. 2 shows that estimates of hSAR_br_ become noisier as cohort size decreases. However, for cohort sizes greater than 1000, and certainly 2000, the variation in estimates is small. The monthly (weekly) cohort size is primarily determined by two factors, namely, the incidence of COVID-19 in the general population, and the threshold *p*_*h*_, i.e. the probability of tweeting about a household member. The later factor is under our control. However the monthly cohort size decreases rapidly with increasing threshold, as discussed in Supplementary Methods S 2 of the Supplementary material. For example, when *p*_*h*_ ≥ 0.05 the total cohort sizes drops from 58,555 to only 8,244.

While “recruitment” of the Twitter cohort is straightforward, cohort size is also determined by the number of index cases tweeting about COVID-19, and thus is strongly related to the community incidence of COVID-19. As noted earlier, when the community incidence is high it is more likely that a second case in the household may have been acquired outside the household. This would have the effect of increasing the estimated hSAR when the cohort size is large. This is observed in Fig. 3A where the curve of hSAR_b_ (no adjustment for probability that source of the secondary infection was outside the household) has a strong correlation (0.7220) with the size of the cohort. We adjust for this by estimating the probability that the source of the second infection was from outside of the household. This is accomplished by randomly pairing Twitter users, assuming homogeneous mixing, considering one as the index and the other as the secondary, and calculating the probability, denoted rSAR, that a random pair will be infected within a 14-day interval. Further details are provided in Section 4.2. As expected, rSAR generally increases with the size of the cohort. After applying this adjustment, the hSAR_br_ estimates have a correlation of 0.504 which is similar to the correlation (0.5304) of UKHSA hSAR estimates and the sizes of their corresponding monthly cohorts.

The average hSAR_br_ value of the period from January 2020 to February 2022 is 0.1598 (CI, 0.1412 to 0.1784). While our hSAR_br_ estimates are usually higher than those of UKHSA, our average is very close to the hSAR estimate of 0.166 (CI 0.140, 0.193) (throughout confidence intervals are 95%) of^28^ based on a meta analysis of 54 relevant studies published until October 2020. A follow-up meta analysis based on 87 studies, published between October 2020 and June 2021, reported an overall SAR of 0.189 (CI 0.162, 0.220)^29^. A further meta-analysis of 63 studies from January 2020 to January 2022 with midpoints through April 2020 reported SAR of 0.155 (CI 0.132-0.182)^30^. A review of studies in the early pandemic phase, dominated by the ancestral strain only, was carried out by^31^ with estimated SAR values ranging from about 0.1 to about 0.45.

During the period under study, there were three primary variants of concern, namely, Alpha, Delta, and Omicron. According to the UK Office of National Statistics^32^, the Alpha variant was dominant from the week ending 18 December 2020 to the week ending 15 May 2021, Delta from the week ending 22 May 2021 to the week ending 19 December 2021 and Omicron from the week ending 23 December 2021 to the week ending 5 September 2022. The average hSAR_br_ during these periods were 0.148 (95% CI, 0.125 to 0.17), 0.154 (95% CI, 0139 to 0.168), and 0.223 (95% CI, 0.202 to 0.245), respectively. A meta-analysis of hSAR by variant^30^ reported corresponding values of 0.364 (95% CI, 0.334 to 0.395), 0.297 (95% CI, 0.230 to 0.373), and 0.427 (95% CI, 0.354 to 0.504). There is generally good qualitative agreement, with small differences in hSAR between Alpha and Delta, and a much larger hSAR for Omicron. Lyngse et al.^33^ analysed 87,677 individuals in 26,675 households in Denmark (restricted to sizes between 2–6 and an average household size of 3.28) during the period 9-22 December 2021 (after contact tracing was stopped, but before the Christmas holidays commenced). During this period Omicron was replacing Delta as the dominant variant. The SAR was estimated as 0.21 for households with a Delta primary case, and 0.29 for households with an Omicron primary case.

Figure 1 provides the dates of commencement (and termination) of primary interventions during the COVID-19 pandemic in England. We observe that the hSAR_br_ declines significantly in the month before the first lockdown in March 2020. It continues to decline until restrictions are eased in June 2020. As expected, the hSAR_br_ then progressively increases. We observe a steady decline in hSAR_br_ beginning in January 2021, when COVID-19 vaccinations were introduced, until May 2021 when hSAR_br_ estimates begin to increase. We note that May 2021 marks the time when the Delta variant becomes dominant. The hSAR_br_ continues to increase until October 2022 which marks the introduction of the third vaccine dose. However, the hSAR_br_ increases after November 2022 as the Omicron variant becomes dominant.

Figure 3B clearly demonstrates that some Twitter users are much more reluctant to disclose the health status of household members. In fact, we estimate that approximately 44% of household COVID-19 infections are not reported. Nevertheless, it is straightforward to estimate the under-reporting and correct for it. An alternative solution might have been to select a cohort that was less reluctant, i.e. to select users for which *p*_*h*_ was significantly greater than 0. However, as discussed earlier, this leads to a very large decline in the size of the monthly cohorts.

We assume in “Methods” that the household size is 2, since the average household size in the UK is 2.3^34^. Our estimates of hSAR are overestimated for household sizes greater than 2 and, conversely, are underestimated for household sizes of 1. On average, we assume that the two effects negate one another as we do not have knowledge of a Tweeter’s household size. If such knowledge is available, or the average household size is higher, e.g. 3, it is straightforward to adjust the Methods accordingly. A further assumption is that a user is only infected once. This is enforced by only taking the single most probable output or output pair from the classifiers. Relaxing this constraint is also straightforward and effectively increases the cohort size, as a user and/or household member will be counted multiple times, once for each period of infection. However, the classifiers are noisy and the increased cohort size comes with the increased risk that positively classified infections are erroneous, since we are essentially lowering the classifier thresholds. To reduce this risk, and because the cohort sizes for each month/week were adequate, we chose to be conservative and only consider the single most likely infection.

We assumed a maximum serial interval of two weeks, i.e. if the two cases are separated by more than two weeks, the cases were treated as independent. The distribution of the serial interval, depicted in Fig. 4A, is almost monotonically decreasing with mean and median values of 6.49 and 6 respectively. While the distribution is similar to that in^35^, the mean/median values are larger than generally reported. A systematic review^36^ of research articles studying the serial interval estimated that the weighted pooled mean serial interval of COVID-19 was 5.2, and a serial interval of 4 days was reported in a study in Spain^37^. Figure 4B-D depict the distribution during the periods when the Alpha, Delta, and Omicron variants were dominant, respectively. The serial intervals for Alpha and Delta are similar with mean values of 6.67 and 6.61. However the distribution for the Omicron variant is clearly different with a mean value of 6.10. While these serial intervals are longer than previously reported elsewhere, they qualitatively support the evidence that the SI for Omicron was shorter than those for the Alpha and Delta variants. The pooled mean serial interval for Delta was estimated to be 3.9 days and Omicron 3.2 days^38^. The UKHSA^39^ has estimated Delta and Omicron serial interval distributions from UK contact tracing data with mean serial intervals of 3.87 days and 3.64 days respectively. Our analysis of the serial interval has at least two potential sources of error. First, we assume that the timestamps associated with the pair of tweets represents the dates of the index and secondary infection. This may not be true - there may be random lags between infection and associated tweet. Further, a tweet of the form “I had COVID 3 days ago” will be classified as the user having COVID-19 at the time the tweet was posted, not 3 days earlier. Further natural language processing could detect and correct for this but remains an avenue for future work.

There are several limitations to this study. At a practical level, Twitter’s new restrictions are an impediment to replication and extensions to our study. However, the impediment is not technological but financial. Twitter’s new policies no longer permit free access to Tweets. Based on Twitter’s current terms and conditions, we estimate that the data collection would now cost between US$700K to US$1.3M. Of course, this is for a period of 26 months. To estimate the hSAR for a single month would cost between US$27K-US$50K.

The study only considered the UK and it may be the case that Twitter behaviour is significantly different in other geographic regions. However, there is evidence^40^ that there is considerable correlation in behaviour across countries, both English speaking (Australia, USA) and non-English speaking. It may be that the classifiers need to be re-trained to identify relevant tweets. However, we note that we obtained sufficient accuracy with a training set of 7894 tweets that were quickly and inexpensively labelled using a crowdsourcing platform. The proposed method may be more useful in low and middle income countries where the conventional health infrastructure needed to support standard epidemiological studies of hSAR is poor or absent, but access to the Web via mobile devices in prevalent. However, verifying the utility of the method is difficult when ground truth data is absent. We further note that the demographics of Twitter users is unlikely to be fully representative of the UK population. It is reported^41^ that there are 25.60 million users in the United Kingdom in early 2024, of which 38.1% are female and 61.9% are male. Data from 2018^42^ states that 33% or users were between the ages of 15 to 24, and that more than half of all users were above the age of 34.

Estimating household secondary attack using classic approaches requires demographic data (household composition) epidemiological data (dates of onset, etc.) and microbiological data (test results). This makes such studies logistically complex, time consuming, and potentially expensive. This new approach opens the potential for hSAR estimates that are cheaper, faster and do not require the collection of data or biological specimens from individuals. With the right calibration and adjustments, our results suggest estimations are within similar ranges as classic methods.

The method is generalizable to other studies. In fact, our work builds on previous work on estimating the hSAR for influenza, as noted earlier. Its appropriateness to other infectious diseases is primarily determined by (i) whether users are inclined/reluctant to tweet about the disease, and (ii) the prevalence of the disease in a population. Thus, for example, it is unlikely to be useful for sexually transmitted diseases where the associated stigma strongly discourages public acknowledgement. Conversely, there have been several studies estimating the prevalence of a variety of infectious diseases, including dengue fever^43^, Zika^44,45^, and Monkeypox^46^ from Twitter, and if prevalence can be estimated, it is likely that the corresponding hSAR can also be estimated.

## Methods

We first describe the data collection and pre-processing steps and then describe the data analysis.

### Data collection

Data collection consists of the following steps:

Step 1: *Identify a seed group*. For the period from January 2020 until March 2022 inclusive, we queried the Twitter API for all tweets that contained keywords or phrase that implied that the tweeter or a household member had COVID-19. The full set of keywords can be found in the supplementary material in Supplementary Methods S 4. Examples of keywords or phrases are “I have covid”, “I have been tested positive for corona”, “husband got covid” and “kid has coronavirus”. Note that the keywords do not, by themselves, define tweets as being about family. For example, a Tweet reading “Joe Biden’s wife’s first name is Jill” contains the keyword “wife” but is not about a family member. Similarly, the absence of a keyword, e.g. “grandmother”, does not imply that there are no tweets containing the word grandmother. For example, a tweet of “My wife is now a grandmother” might be included in the training set since the tweet contains the keyword “wife”. Note further, that the keywords are only used to construct the training set, which is subsequently manually labelled. During training, the classifiers are free to select and weight any words to optimise performance. This query resulted in a total of 2,001,896 tweets from 1,226,509 unique seed users.
Step 2: *Geolocation*. The 1,226,509 unique seed users can be located anywhere in the world. We therefore applied geolocation filtering to retain only users whose tweets originate from the UK.
  To identify if a user tweeted from the UK, we collect the user information of each user. This is publicly available information associated with a Twitter user account, and consists of several fields, including *‘user creation timestamp’, ‘user description’*, and *‘location’*. If a Twitter user’s description is unavailable, we delete the user. This reduced the number of unique users from 1,226,509 to 1,145,503.
  We perform a keyword lookup in the *user description* and the *location* fields to identify if the user belongs to the UK. We use a list of the top 20 most populated cities in the UK^47^ as well as additional keywords. The additional keywords used are {*‘England’, ‘Scotland’, ‘Wales’, ‘Northern Ireland’, ‘United Kingdom’, ‘UK’, ‘Newport’, ‘Belfast’, ‘Derry’*}. This list includes four regions of the UK and the top two most populated cities in these regions.
  Many of the 23 cities in the United Kingdom have the same name as cities in the United States or elsewhere. To disambiguate city names, a user is considered to be tweeting from UK only if the city name (that is same in the UK and any other country) is accompanied by one of the keywords from the list {*‘England’, ‘United Kingdom’, ‘UK’*}. Note that 15 cities (Bristol, Leeds, Preston, Liverpool, London, England, Islington, Reading, Sheffield, Birmingham, Leicester, Manchester, Coventry, Nottingham, Sunderland) required disambiguation. For example, *‘Liverpool’* is a city in both the UK and the US. We mark it as UK location only if it is in one of the following forms {*‘Liverpool, England’, ‘Liverpool, United Kingdom’, ‘Liverpool, UK’*}. Even though *‘London’* is located in the UK, US and Canada, we always mark it as a UK location because of its high population in the UK.
  After geofiltering, the number of unique users is reduced from 1,145,503 to 77,016.
Step 3: *Download users’ timelines*. After geofiltering, we downloaded the most recent 3200 tweets for each of the 75,440 users. Note that this is less than the 77,016 remaining in the previous step, and reflects that some users (1576) were no longer available. Note that 3200 is an arbitrary number determined by the Twitter API. Also note, that some users will have less than the maximum number of 3200 tweets.
Step 4: *Filtering Twitter Cohort*. Collecting the set of seed tweets and corresponding seed users occurred some months before we collected users’ timelines.(This delay was in part due to staff interruptions and the COVID-19 pandemic.) We observed that almost 19% of users’ timelines did *not* include the original seed tweet. There are at least two possible reasons for the absence of a seed tweet. First, the user may have deleted the tweet. Second, for prolific tweeters, the duration of the 3200 timeline may not encompass the seed. We decided to remove all users whose seed tweet was not found in their time line. There were 13,010 prolific users (97.24%) out of 13,379 users not containing the seed tweets. This reduced the number of unique users from 75,440 to 62,061.
Step 5: *Filtering user’s timeline*. We apply the Household classifier (C3) to the timelines of each of the 62,061 users to determine each user’s probability of tweeting about a household member. We remove all users who never tweet about a household member. This reduces the number of unique users from 62,061 to 58,555. More generally, we can set a threshold on the probability, *p*_*h*_, of tweeting about a household member and remove all users whose probabilities are less than *p*_*h*_.
Step 6: *Identifying a user’s COVID-19 tweet(s)* We assume that a user is only infected once. However, usually there are multiple references to infection in a user’s timeline. The following process was used to filter a user’s timeline to a single pair of tweets, one indicating that the user had COVID-19 and the other that a household member was infected, or a single tweet indicating that the user or household member was infected.

We constructed three classifiers, see Supplementary Methods S 6 and 7 for details, to independently identify whether a tweet was (i) about the user/Tweeter having COVID-19, (ii) about a household member having COVID-19, and/or (iii) about a household member (irrespective of topic), denoted C1, C2, and C3, respectively. The classifiers were constructed based on a labelled dataset of 7894 tweets created using a crowdsourcing platform. Each tweet was labelled by three labellers. For all cases where there was disagreement between labellers, the labellers were required to resolve the discrepancies. The best performing classifiers, based on 10-fold cross validation, used CT-BERT^48^, and resulted in accuracies of 0.8886, 0.9324 and 0.7839 for the classifier C1, C2, and C3, respectively. The AUCs for the three classifiers are 0.907, 0.934, and 0.768, respectively. Note that for a tweet to be classified as about a household member having COVID-19, it must also be positively classified by both C2 and C3, i.e. the tweet must be classified as both “about a household member having COVID-19” and “about a household member”. Clearly, if this is not the case, one of the classifiers is wrong, but which classifier is incorrect is unknown. In such cases, we conservatively chose to ignore the positive classification.

For each user, *u*, we determine the sets of tweets {*C*1_*u*_} and {*C*2_*u*_} that exceed the classifiers’ thresholds. {*C*1_*u*_} is the set of Tweets from user *u* that were positively classified as being about the user, *u*, having COVID-19. Similarly, {*C*2_*u*_} is the set of Tweets from user *u* that were positively classified as being about the user, *u*’s, household members having COVID-19. Each set may have 0, 1 or more entries.

We observed that each set may contain almost identical tweets, e.g. a retweet of a previous tweet. To remove these copies we performed the following steps:

1. We removed (i) all user-mentions starting with ‘@’, (ii) hyperlinks or website links starting with ‘https’ or ‘www’, and (iii) replaced all emojis with their textual equivalent using Python package *emoji.demojize*. All that remains is alphanumeric text.
2. Tweets with identical text were then identified and only the earliest tweet is retained.

This results in two filtered sets $\{C{1}_{u}^{{\prime} }\}$ and $\{C{2}_{u}^{{\prime} }\}$. The classifier score for each tweet, *i*, in these sets is then converted to a “probability” with values between 0 and 1 using the equation

1 $$P1(u,i)=\frac{1}{2}+\frac{1}{2}\cdot \frac{C1(u,i)-{\tau }_{1}}{\max (C1)-{\tau }_{1}}$$

2 $$P2(u,j)=\frac{1}{2}+\frac{1}{2}\cdot \frac{C2(u,j)-{\tau }_{2}}{\max (C2)-{\tau }_{2}}$$

where, *C*1(*u*, *i*) and *C*2(*u*, *j*) are the classification scores for C1 and C2 for tweet *i* or *j* of user *u*. The classifier thresholds are denoted by *τ*_1_ and *τ*_2_. The function max() returns the maximum score across all users from the classifier passed to it.

Next, we consider all $\{C{1}_{u}^{{\prime} }\}\times \{C{2}_{u}^{{\prime} }\}$ pairs of tweets. All tweets include their corresponding date of publication (Timestamp). We remove pairs where the time between them exceeds a specified threshold (two weeks). The remaining pairs, if any, are ranked according to the product of their probabilities. Only the top-ranked pair is retained. This pair, if it exists, is given the date of the earliest of the two tweets.

In the case where there is no pair, there are two possible scenarios:

- Either the set {$C{1}_{u}^{{\prime} }$} or {$C{2}_{u}^{{\prime} }$} is empty. In this case, the user is assigned to the category of the non-empty set.
  - If {$C{1}_{u}^{{\prime} }$} is not empty, the tweet with the maximum *P*1 is selected
  - If {$C{2}_{u}^{{\prime} }$} is not empty, the tweet with the maximum *P*2 is selected
- All pairs in $\{C{1}_{u}^{{\prime} }\}\times \{C{2}_{u}^{{\prime} }\}$ have a time difference of more than two weeks. In this case, the two tweets with the maximum *P*1 and *P*2 are retained.

### Analysis

For a given month (week), we determine the set of users, *A*, who tweeted that they and/or a household member had COVID-19 in this month. The set *A* is the union of *A*_1,0_ (those that only tweet about themselves), *A*_2,0_ (only about household members), *A*_1,2_ (those that tweet about themselves and then household members), and *A*_2,1_ (household members then themselves).

Consider the two cases *A*_1_ = *A*_1,0_ ∪ *A*_1,2_, i.e. the subset of users who only tweet about themselves having COVID-19 or who tweet that they have COVID-19 before tweeting about a household member having COVID-19, and *A*_2_ = *A*_2,0_ ∪ *A*_2,1_, i.e. the subset of users who only tweet about a household member having COVID-19 or who tweet that a household member has COVID-19 before tweeting about themselves having COVID-19. Let *a*_1_ and *a*_2_ denote the actions of tweeting about the user or a household member having COVID-19, respectively. Then the probability that individual, *i*, will tweet about a household member having COVID-19 after tweeting that they have COVID-19 is

3 $${P}_{i}({a}_{2}| {a}_{1})=\left(1-{(1-{\alpha }_{1})}^{n-1}\right){P}_{i}({a}_{2}| {E}_{2},{a}_{1})$$

where *E*_2_ represents the event that a household member has COVID-19, and $\left(1-{(1-{\alpha }_{1})}^{n-1}\right)$ represents the probability that a household member will get infected given *α*_1_ is the secondary attack rate and *n* is the size of the household. The average household size for UK is *n* = 2.3, so (*n* − 1) = 1.3 ≈ 1, which gives us

4 $${P}_{i}({a}_{2}| {a}_{1})={\alpha }_{1}{P}_{i}({a}_{2}| {E}_{2},{a}_{1})$$

As in^26^, we assume that *P*_*i*_(*a*_2_∣*E*_2_, *a*_1_) is equal to *P*_*i*_(*a*_3_), where, *a*_3_ denotes the action of user *i* tweeting about a household member, irrespective of the subject.

Summing over all *m*_1_ users in *A*_1_, and dividing both sides by *m*_1_, we have

5 $$\frac{1}{{m}_{1}}\sum\limits_{i-1}^{{m}_{1}}{P}_{i}({a}_{2}| {a}_{1})=\frac{{\alpha }_{1}}{{m}_{1}}\sum\limits_{i=1}^{{m}_{1}}{P}_{i}({a}_{3})$$

The LHS is simply the average probability, *P*(*a*_2_∣*a*_1_) of tweeting about a household member with COVID-19 after tweeting that the user has COVID-19, and can be empirically estimated as,

6 $$P({a}_{2}| {a}_{1})=\frac{| {A}_{1,2}| }{| {A}_{1,2}| +| {A}_{1,0}| }$$

The SAR, *α*_1_, estimated from the cohort subset *A*_1_ is then

7 $${\alpha }_{1}=\frac{| {A}_{1,2}| }{P({a}_{3})\times \left(| {A}_{1,2}| +| {A}_{1,0}| \right)}$$

where

8 $$P({a}_{3})=\frac{1}{{m}_{1}}\sum\limits_{i=1}^{{m}_{1}}{P}_{i}({a}_{3})$$

Similarly for the subset *A*_2_, since we assume that the household size *n* = 2, the probability that user, *i*, tweets about having COVID-19 after tweeting that a household member has COVID-19 is simply

9 $${P}_{i}({a}_{1}| {a}_{2})={\alpha }_{2}{P}_{i}({a}_{1}| {E}_{1},{a}_{2})$$

where *α*_2_ is the secondary attack rate, and *E*_1_ denotes the event that the users has COVID-19. Since user *i* has already tweeted that a householder has COVID-19, we assume that *P*_*i*_(*a*_1_∣*E*_1_, *a*_2_) = 1. Note that this probability is likely to be less than 1, so this is a lower bound on the secondary attack rate. Summing Equation (9) over all users, *m*_2_, and rearranging, we get

10 $${\alpha }_{2}=\frac{1}{{m}_{2}}\sum\limits_{i=1}^{{m}_{2}}{P}_{i}({a}_{1}| {a}_{2})$$

The right hand side of Equation (10) is the average probability, *P*(*a*_1_∣*a*_2_), which can be empirically estimated as

11 $${\alpha }_{2}=\frac{| {A}_{2,1}| }{| {A}_{2,1}| +| {A}_{2,0}| }$$

The overall SAR estimate, *α*, is a weighted average of *α*_1_ and *α*_2_, i.e. *α* = *α*_1_*w*_1_ + *α*_2_*w*_2_ where *w*_1_ and *w*_2_ are given by Equation (12), respectively.

12 $$\begin{array}{rcl}{w}_{1}&=&\frac{| {A}_{1,2}| +| {A}_{1,0}| }{| {A}_{1,2}| +| {A}_{1,0}| +| {A}_{2,1}| +| {A}_{2,0}| }=\frac{| {A}_{1,2}| +| {A}_{1,0}| }{| A| }\\ {w}_{2}&=&\frac{| {A}_{2,1}| +| {A}_{2,0}| }{| {A}_{1,2}| +| {A}_{1,0}| +| {A}_{2,1}| +| {A}_{2,0}| }=\frac{| {A}_{2,1}| +| {A}_{2,0}| }{| A| }\end{array}$$

This results in Equation (13) for SAR approximation.

13 $${{{{\rm{hSAR}}}}}_{{{{\rm{b}}}}}=\frac{P({a}_{3})\cdot | {A}_{2,1}| +| {A}_{1,2}| }{P({a}_{3})\cdot \left(| {A}_{2,1}| +| {A}_{2,0}| +| {A}_{1,2}| +| {A}_{1,0}| \right)}$$

When *P*(*a*_3_) = 1, Equation (13) provides a lower bound for the SAR scores.

#### Adjustment for reluctance to tweet about household members

Equation (13) includes an adjustment, *P*(*a*_3_) to account for user reluctance to tweet about a household member. This probability should be empirically estimated via Equation (8). However, in practice, the individual probabilities, *P*_*i*_(*a*_3_) are quite small and the resulting SAR estimates were unstable with values sometimes exceeding 1. To resolve this issue, we considered the two independent estimates of hSAR given by *α*_2_ and *α*_1_. We assume a linear relationship, i.e.

14 $${\alpha }_{1,t}=g{\alpha }_{2,t}+c$$

where *t* denotes time in months or weeks. If there was no reluctance to tweet about a household member, we expect *g* = 1 and *c* = 0. In practice, the empirical gradient is 0.56 and *c* = 0.06. We ignore the bias, *c*, and set *P*(*a*_3_) = *g*. See Supplementary Methods S 1 of the Supplementary material for further details.

#### Adjustment for infection outside of the household

The fact that a user and a household member both acquire COVID-19 within a given serial interval does not preclude the probability that the sources of both infections may be outside the home. This probability increases as the prevalence of the disease in the community increases. We accounted and enumerated for this as follows.

For the given cohort (*n* = 58, 555) we created *n*/2 random pairs assuming homogeneous mixing. The first member of the pair is considered the user (primary infection) and the second member the household member (secondary infection). The monthly estimation of SAR proceeds as before where the first member’s tweets about themselves having COVID-19 are paired with the second member’s tweets about themselves having COVID-19, i.e. the output from the classifier C2 is *not* used. The resulting values, denoted rSAR, are an estimate of the probability that the secondary infection was acquired outside of the household. These monthly (weekly) estimates are subtracted from our estimate hSAR_b_ to produce our final adjusted estimate, hSAR_br_

15 $${{{{\rm{hSAR}}}}}_{{{{\rm{br}}}}}={{{{\rm{hSAR}}}}}_{{{{\rm{b}}}}}-\,{{\mbox{rSAR}}}\,$$

### Inclusion and ethics statement

This research has been conducted in accordance with ethical standards and principles. Approval for the study protocol, including the collection, analysis, and publication of data, was obtained from the UCL Research Ethics Committee (REC), (i) UCL REC 16621/003 “Estimating the secondary attack rate and serial interval of COVID-19 using Twitter” and (ii) the UCL Computer Science REC /CSREC/R/30 “Estimating the secondary attack rate and serial interval of COVID-19 using X, formerly known as Twitter”. Given the large size of the Twitter cohort, obtaining informed consent from all participants involved in the study was not feasible. This exception was approved by the ethics committees at UCL REC 16621/003 and UCL/CSREC/R/30.

### Reporting summary

Further information on research design is available in the Nature Research Reporting Summary linked to this article.

### Supplementary information

Supplementary material

Reporting Summary

## Data Availability

Twitter only permits up to a maximum of 1,500,000 Tweet IDs to be distributed. Due to this limitation, we only provide the Tweet ID’s of each of the tweets that are positively classified by the three classifiers. We also provide the labelled data used to train the three classifiers. This is a total of 460,979 ID’s. Data is available at https://figshare.com/s/375a15bbcca69af95822.

## Code availability

The code for this study is publicly available at https://github.com/AarzooDhiman/COVID_SAR. The code is licensed under a Creative Commons Attribution-NonCommercial 4.0 International License (CC BY-NC 4.0).
