# Supplementary material for: Estimating the household secondary attack rate and serial interval of COVID-19 using social media
Source: NPJ Digit Med. 2024 Jul 20;7:194. doi: 10.1038/s41746-024-01160-2 (PMC11271293; doi:10.1038/s41746-024-01160-2)
Supplement: Supplementary file 1 — Supplementary material [file 41746_2024_1160_MOESM1_ESM.pdf]

Supplementary Material

April 25, 2024

1 Supplementary Methods

S 1 Reluctance to tweet about the household member

Supplementary Figure 1 shows a scatter plot of corresponding values of α_1 and α_2 and the best line fit.

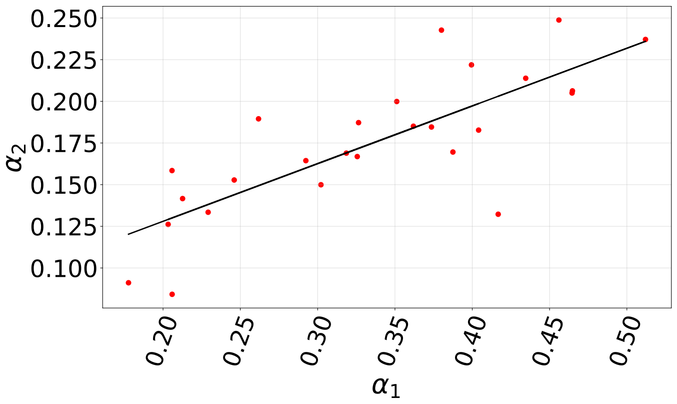

Supplementary Figure 1: A scatter plot of α_1 versus α_2 and the fitted straight line.

S 2 Cohort Size and hSAR when $p_h \geq 0.05$

Supplementary Figure 2 shows the total number of users with a probability of tweeting about a household member (irrespective of topic) greater than or equal to p_h.

For $p_h \geq 0.05$ Supplementary Figure 3 shows the associated monthly cohort sizes and the corresponding estimates for hSAR_{br}.

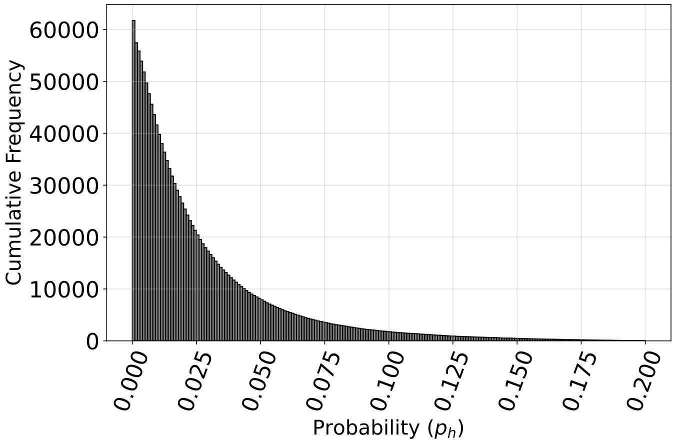

Supplementary Figure 2: Histogram (bin width = 0.001) of the number of users with a probability, p_h, that a user will tweet about a household member, greater than a threshold x, for $0 \leq x \leq 0.2$.

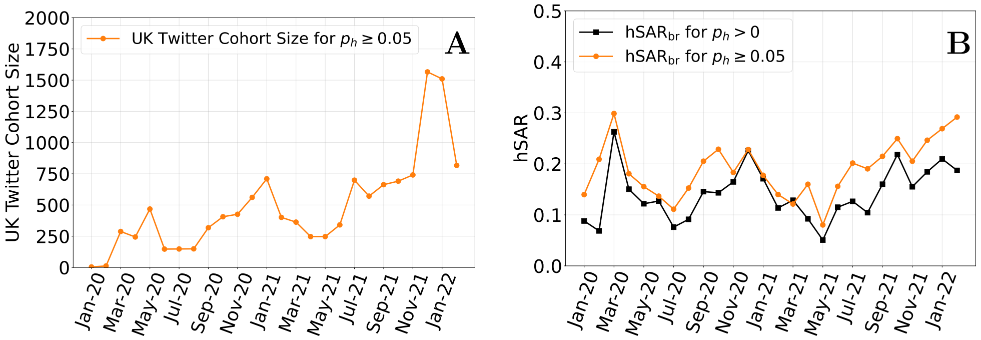

Supplementary Figure 3: **A.** The monthly size of the Twitter cohort when including only users with a probability of tweeting about a family member greater than 0.05 ($p_h \geq 0.05$). **B.** The corresponding household adjusted hSAR ($hSAR_{br}$) assuming a maximum serial interval of 14 days.

S 3 Comparison to UKHSA hSAR estimates

We compare our estimations using the hSAR values published by UKHSA. We use the technical briefings provided by UKHSA on the investigation of SARS-CoV-2 variants[1]. These reports provide an estimate of monthly hSAR values for COVID-19 starting from month Jan 2021. These reports calculate hSAR as the number of household contacts becoming cases divided by all household contacts in a given time period from Jan 2021 to Nov 2021 (see Supplementary Equation Supplementary Equation 1). For months Dec 2021 to Feb 2022, they provide the SAR scores adjusted to vaccination status of the exposer and the contact (allowing for interaction with variant), age and sex of the exposer and the contact, the date (week) of positive test of the exposer and whether the contact completed contact tracing[2]. For our comparison, we only use the numbers given for the household contacts. As given in Supplementary Equation Supplementary Equation 1, V is the set of variants used for calculation. For our calculations, we used five variants i.e. Alpha, Delta, Omicron, Delta plus, Omicron BA.2. Supplementary Figure 4 provides the un-adjusted PHE provided SAR scores, that shows a huge peak in Nov 2021, without adjustment. All other figures show the adjusted PHE SAR scores.

[1] https://www.gov.uk/government/publications/investigation-of-sars-cov-2-variants-technical-briefings

[2] https://assets.publishing.service.gov.uk/government/uploads/system/uploads/attachment_data/file/1060337/Technical-Briefing-38-11March2022.pdf

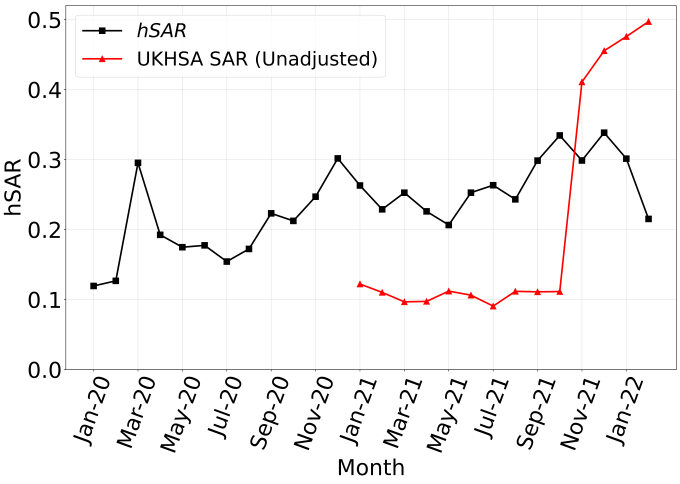

Supplementary Figure 4: The monthly household SAR (hSAR) calculated using the Twitter cohort consisting of users with a probability of tweeting about a family member greater than zero ($p_h > 0$) and a maximum of 14 days interval in both the infections. The UKHSA hSAR (unadjusted) is the weighted average of the SAR scores for different variants published by UKHSA by using only the raw numbers given for the number of cases among contacts and the total number of contacts, i.e. not using the unadjusted SAR scores given by PHE.

$$\text{UKHSA SAR (Unadjusted)} = \sum_{v=\{v_1,v_2,\ldots v_n\}} W_v \cdot S_v$$

$$W_v = \frac{\text{Cases}_v}{\sum_{v=\{v_1,v_2,\ldots v_n\}} \text{Cases}_v} \qquad \text{Supplementary Equation 1}$$

$$S_v = \frac{\text{Cases}_v}{\text{Contacts}_v}$$

where v_i represents variant i.

S 4 List of seed phrases

Below is a list of the seed phrases used in Step 1 of Section 4.1 to identify a seed group of Twitter users.

Author-related phrases:

"i have covid" OR "i might have covid" OR "i had covid" OR "ve got covid" OR "i got covid" OR "ve had covid" OR "i tested positive for covid" OR "ve tested positive for covid" OR "ve been tested positive for covid" OR "I been tested positive for covid" OR "i have corona" OR "i might have corona" OR "i had corona" OR "ve got corona" OR "i got corona" OR "ve had corona" OR "i tested positive for corona" OR "ve tested positive for corona" OR "ve been tested positive for corona" OR "I lost my sense of smell" OR "ve lost my sense of smell" OR "I lost my sense of taste" OR "ve lost my sense of taste" OR "I lost sense of smell" OR "ve lost sense of smell" OR "I lost sense of taste" OR "ve lost sense of taste" OR "I lost the sense of smell" OR "ve lost the sense of smell" OR "I lost the sense of taste" OR "ve lost the sense of taste" OR "I lost taste and smell" OR "ve lost taste and smell" OR "I lost smell and taste" OR "ve lost smell and taste"

Family-related phrases:

("husband" OR "wife" OR "partner" OR "daughter" OR "son" OR "mum" OR "mom" OR "mommy" OR "dad" OR "parent" OR "mate" OR "boyfriend" OR "girlfriend" OR "kid" OR "child") ("has covid" OR "might have covid" OR "had covid" OR "got covid" OR "tested positive for covid" OR "has corona" OR "might have corona" OR "had corona" OR "got corona" OR "tested positive for corona" OR "has coronavirus" OR "might have coronavirus" OR "had coronavirus" OR "got coronavirus" OR "tested positive for coronavirus" OR "sense of smell" OR "sense of taste" OR "lost smell" OR "lost taste")

S 5 Classifiers

Supplementary Section S 5.1 briefly describes the training data. Supplementary Section S 5.2 describes the preprocessing steps applied to each tweet prior to input to the classifier, which is described in Supplementary Section S 5.3.

S 5.1 Training Data

The three classifiers, C1, C2 and C3, were trained on a set of 7894 Tweets. Each tweet has been given a *True* or *False* label for each of the following five mutually non-exclusive categories:

L1 Is the tweet about the author having a COVID-19 infection?

L2 Is the tweet about author having COVID-19 related symptoms?

L3 Is the author writing about a family member?

L4 Is the author writing about a family member having COVID-19 infection?

L5 Is the author writing about a family member showing COVID-19 symptoms?

The percentage of tweets in each of the categories L1, L2, L3, L4, and L5 are 19.72%, 10.98%, 26.17%, 14.22% and 4.53% , respectively.

The categories L1 and L2 were used as positive examples for classifier C1, which identified tweets indicating that the user had COVID-19. Similarly, the categories L4 and L5 were used as positive examples for classifier C2, which identified tweets indicating that a household member had COVID-19. Finally category L3 was used as positive examples for classifier C3 which identified tweets about a household member, irrespective of topic.

Details of the labeling process are described in Supplementary Section S S 6.

S 5.2 Data pre-processing

Tweets consist of unstructured text. All tweets were preprocessed such that (i) all contractions were expanded, (ii) all non-alphabetic characters, except hashtag (#) were removed, (iii) character repetitions of three or more were replaced with two, e.g. "heeelp" becomes "heelp", and (iv) emojis were replaced by their textual descriptions as defined in the Python package *emoji.demojize*. Note that we did not perform stemming or stopword removal.

S 5.3 Classifier

A tweet can belong to more than one class. We trained two separate classifiers, classifier C1 which predicts if the author is talking about himself having a COVID-19 infection or symptoms and classifier C2 which predicts if the author is talking about a family member having a COVID-19 infection or symptoms. The classifier C1 is trained on the union of two sets of labels (i) Author infected with COVID-19 (L1) and (ii) Author having symptoms of COVID-19 (L2) (containing 25.76% of True samples). Classifier C2 is trained on the union of the two sets of labels (i) Family member infected with COVID-19 (L4) and Family member having symptoms of COVID-19 (L5) (containing 16.25% of True samples). Classifier C3 predicts if the tweet is about a family member and uses the label set L3 (containing 26.17% of True samples). Classifier C1, C2 and C3 follow a similar architecture as given in Supplementary Figure 5.

The input to our classifiers are tweet texts. The tweet texts are required to be represented in the form of numerical vectors to be fed into any classification model.

We use the pre-trained language model Covid-Twitter BERT(version 2) (CT-BERT[3]) [4], a sub-domain specific fine-tuned version of the BERT-large-uncased model [1]. CT-BERT was trained on 22.5M preprocessed COVID-19 related tweets (40.7M sentences and 633M tokens) collected between January 12 and April 16, 2020 containing at least one of the keywords "wuhan", "ncov", "coronavirus", "covid", or "sars-cov-2". The CT-BERT model has been trained on inputs with a sequence length of 96 tokens. We use a sequence length of 60 tokens in place of 96 tokens used originally in CT-BERT. We used 60 tokens because 94.68% of the tweets had lengths less than or equal to 60 as shown in

[3]https://huggingface.co/digitalepidemiologylab/covid-twitter-bert-v2

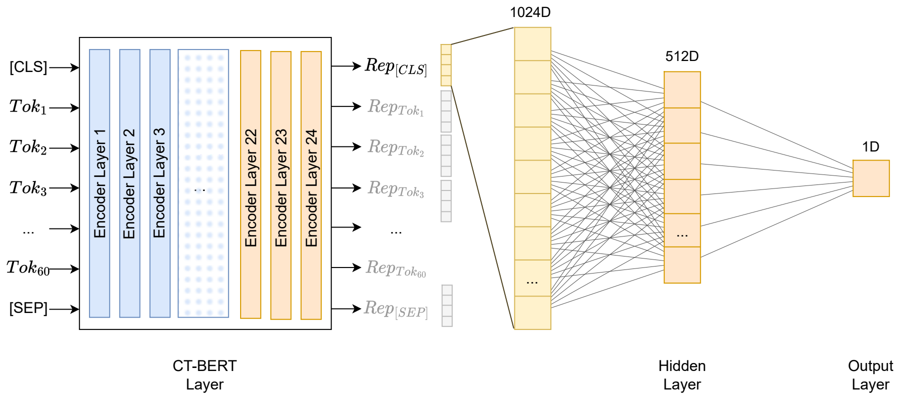

Supplementary Figure 5: The CT-BERT neural network classifier architecture. In the proposed model, the input token sequence undergoes two distinct stages: the CT-BERT layer, which consists of a combination of fixed neural network layers (shown in blue) and the last three neural network layers that are fine-tuned (shown in orange). The CT-BERT layer extracts embeddings from the input tokens, with the [CLS] token's embedding being selected as the representation for the downstream task. The chosen [CLS] token embedding of 1024 dimensions, serves as the input to a subsequent dense neural network model. This neural network contains a hidden layer with 512 dimensions. Finally, the output layer of this neural network consists of a single neuron, for the classification.

Supplementary Figure 6. We fine tune CT-BERT model on our labeled data for classifier C1, C2 and C3. We use the same set of tweets for C1, C2 and C3 only changing the target labels of each tweet for the respective classifier.

The BERT Tokenizer[4] was used to generate the input sequence (size: $[n \times 60]$ and input mask (size: $[n \times 60]$) where n is the number of training samples. The input sequence, input mask and target labels are then partitioned into batches of size 32. The parameters of the combined neural network classifier are tuned after each batch.

The CT-BERT encoder produces a sequence of hidden states for each token in one input sequence. However, for the classification task we only need a single vector. A special input token [CLS] is appended at the start of each input sequence and [CLS] is also masked during the pertaining and fine-tuning task. The vector representation generated for the [CLS] token is used as the sentence representation (tweet representation in our case). It is a vector of 1024 dimensions, equal to the size of the hidden state.

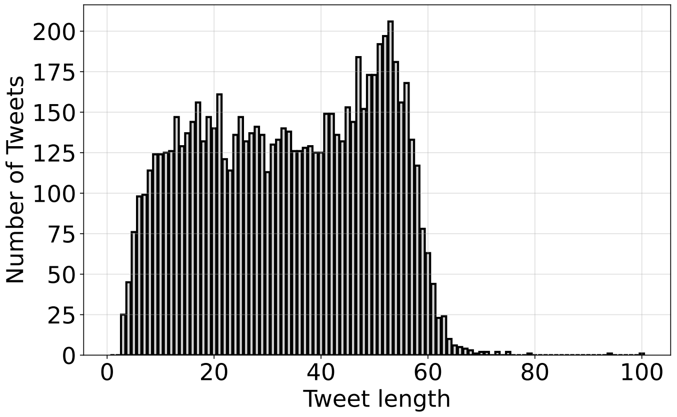

Supplementary Figure 6: Histogram plot representing count of tweets for a given tweet length.

We fix the first 21 (of 24) encoder layers in CT-BERT, i.e. we only train the last three encoder layers on our data. It was observed that there was an insignificant change in the performance of the

[4]https://huggingface.co/docs/transformers/main_classes/tokenizer

classifier after fine-tuning CT-BERT beyond three encoder layers. This is consistent with [3].

The sentence representation ([CLS]) is then fed to the simple feed-forward architecture that has an input layer of size 1024 neurons, followed by a hidden layer of size 512 neurons, and an output layer of 1 neuron. We use a Leaky Relu activation function with a negative slope of 0.1 on the hidden layer. To reduce overfitting in the neural network, caused by the small number of training samples, a dropout layer of rate 0.5 is added to the network during training. The hidden layer outputs are forwarded to the output layer which uses the Sigmoid activation function. The Sigmoid activation function guarantees the single output lies in the range 0 to 1.

For the 3 layer feedforward network, we use a learning rate decay starting from 2×10^{-5} with a factor of 0.01 [2]. The optimizer used is $AdamW$ with a weighted binary cross entropy loss function

$$l = \frac{1}{N}\sum_{i=1}^{N}(p_c y_i \log(x_i) + (1 - y_i)(1 - \log(x_i))) \qquad \text{S2}$$

where y_i is the target label either 0 or 1, x_i is the input i.e. the raw output of the single output layer neuron, p_c is the weight used to adjust for the positive examples, and is the ratio of the negative to positive samples in the target, i.e., the more negative samples there are, the greater the weight adjustment on positive samples, and N is the batch size.

For each classifier, we used 10-fold cross-validation with stratified folds, i.e. each mutually exclusive fold has the same ratio of positive to negative examples. We maintain the ratios of 25:75, 16:84 and 26:74 for classifier C1, C2 and C3, respectively, that is the original proportion of positive examples used for classifiers C1, C2 and C3. We use the training and validation sets to tune the parameters and the test set to evaluate the performance of the classifier. Since the output layer yields a value between 0 and 1 as the prediction score, we derive a threshold on the prediction score such that it maximizes the F1 score of the validation set. For a given classifier, for each test set, there are 9 training and validation sets. The thresholds obtained from the 9 validation sets are averaged to derive a threshold for the test set. The average threshold is used to assign labels to the test set. The predicted labels for each test set are merged to evaluate the overall performance of the classifier. These steps are repeated for all three classifiers.

The classifier accuracies were 0.8886, 0.9324 and 0.7839 and F1-scores of 0.8538, 0.8733 and 0.7202 for the classifiers C1, C2, and C3 respectively. The AUC ROC for the three classifiers are 0.907, 0.934, and 0.768, respectively. For comparison, a majority-class classifier, which labels all tweets as negative, has accuracies 0.7410, 0.8366 and 0.7377 and F1-scores of 0.4254, 0.4555 and 0.4245 respectively.

S 6 Label Task instructions

The following instructions were given to Labelbox.

For each displayed text, you will have a Date and a Statement. The Date follows the format: YYYY-MMDD. Consider the sentence is being said at the given date. Your task is to answer five ”yes/no” questions. The questions are as follows:

Is the text about...

1. the author having COVID-19?
2. the author having symptoms of COVID-19?
3. a member of the author’s household?
4. a member of the author’s household having COVID-19?
5. a member of the author’s household having symptoms of COVID-19?

Rules to follow while labeling the data

- A sentence should mention the COVID-19 infection or COVID-19 symptoms. If the sentence shows the author is in doubt regarding his/her COVID-19 infection or symptom then this should be treated as a negative statement. For example,

  - “I’m sure I have covid”, “I contracted covid” are **YES**
  - “I’m worried I might have covid”, “I think I might have covid” are **NO**
  - “I tested positive for covid” is **YES**
  - “I’m getting tested for covid tomorrow”, “I’m worried my test will come back positive” are **NO**

- There may be some statements which mention the time of the COVID-19 infection. We are interested in texts that indicate that the author or household member has COVID-19 at the time of writing (as provided in the date field) or withing three weeks of writing.

- Below are the descriptions of the following:

  - ‘My mother got tested positive yesterday’ – **YES**
  - ‘I tested positive for covid a week ago’, – **YES**
  - ‘My mother tested positive for covid two months ago and she is still recovering’ – **NO**
  - *Date: 20 March 2020 Statement:* ‘I tested positive in January and then my mother got infected in February.’ Here it is a **NO** for the AUTHOR COVID (because the author was infected 2 months ago), but **YES** for FAMILY COVID (because the author’s mother got infected 1 month ago, which could just be within the 3 week period).
  - “My experience with COVID-19 was horrible. I could not breathe properly.”- **YES**
  - “My experience with COVID-19 last year was horrible. I had to go to hospital.”- **NO**

Most common Covid symptoms

- high temperature
- cough
- loss of smell or taste (formal terms: anosmia or ageusia)
- fatigue and overall bad feeling

Who counts as a member of an author’s household?
People likely to live together. e.g. partners, wife, husband, parents, children, siblings, flatmates, roommates. Not friends or neighbours.

S 7 Example Labels

EXAMPLE 1:
Date: 2021-06-23
Text: ***“It’s my baby’s birthday!”***
Is the text about...

1	Does the author have COVID-19?	**No**
2	Does the author have symptoms of COVID-19?	**No**
3	Is a member of the author’s household or family?	**Yes**
4	Does a member of the author’s household or family have COVID-19?	**No**
5	Does a member of the author’s household or family have symptoms of COVID-19?	**No**

Why? – Because in this statement the author is talking about his/her child (family)

EXAMPLE 2:
Date: 2021-03-01
Text: ***“My mum has been diagnosed with Covid. I am so worried about her”***
Is the text about...

1	Does the author have COVID-19?	**No**
2	Does the author have symptoms of COVID-19?	**No**
3	Is a member of the author's household or family?	**Yes**
4	Does a member of the author's household or family have COVID-19?	**Yes**
5	Does a member of the author's household or family have symptoms of COVID-19?	**No**

Why? – Because in this statement the author talks about his mother having Covid infection.

EXAMPLE 3:
Date: 2020-04-09
Text: *"Should I be worried if my hubby lost his sense of smell?"*
Is the text about...

1	Does the author have COVID-19?	**No**
2	Does the author have symptoms of COVID-19?	**No**
3	Is a member of the author's household or family?	**Yes**
4	Does a member of the author's household or family have COVID-19?	**No**
5	Does a member of the author's household or family have symptoms of COVID-19?	**Yes**

Why? – Because in this statement the author talks about his husband having Covid symptom.

EXAMPLE 4:
Date: 2020-07-18
Text: *"I'm not feeling well so I'm getting tested for covid. Wish me luck!"*
Is the text about...

1	Does the author have COVID-19?	**No**
2	Does the author have symptoms of COVID-19?	**Yes**
3	Is a member of the author's household or family?	**No**
4	Does a member of the author's household or family have COVID-19?	**No**
5	Does a member of the author's household or family have symptoms of COVID-19?	**No**

Why? – Because the author may or may not have covid but has some unknown covid symptoms.

EXAMPLE 5:
Date: 2020-10-02
Text: *"Me and my partner woke up today with all coronavirus symptoms"*
Is the text about...

1	Does the author have COVID-19?	**No**
2	Does the author have symptoms of COVID-19?	**Yes**
3	Is a member of the author's household or family?	**Yes**
4	Does a member of the author's household or family have COVID-19?	**No**
5	Does a member of the author's household or family have symptoms of COVID-19?	**Yes**

Why? – The author talks about him and his partner having covid symptoms.

EXAMPLE 6:
Date: 2021-06-12
Text: *"My neighbour always complains about her daughter's performance at school"*
Is the text about...

1	Does the author have COVID-19?	**No**
2	Does the author have symptoms of COVID-19?	**No**
3	Is a member of the author's household or family?	**No**
4	Does a member of the author's household or family have COVID-19?	**No**
5	Does a member of the author's household or family have symptoms of COVID-19?	**No**

Why? – This is not about the author or their family.

EXAMPLE 7:
Date: 2020-11-03
Text: ***"I had all these symptoms 3 weeks ago. Put it down to man flu. The fatigue was the worse. Did I have Covid. I don't know. Wife had before me"***
Is the text about...

1	Does the author have COVID-19?	**No**
2	Does the author have symptoms of COVID-19?	**Yes**
3	Is a member of the author's household or family?	**Yes**
4	Does a member of the author's household or family have COVID-19?	**No**
5	Does a member of the author's household or family have symptoms of COVID-19?	**No**

Why? –The author mentions that he got covid 3 weeks ago, which is within the time of interest.

EXAMPLE 8:
Date: 2020-11-03
Text: ***"I had all these symptoms in October. Put it down to man flu. The fatigue was the worse. Did I have Covid. I don't know. Wife had before me."***
Is the text about...

1	Does the author have COVID-19?	**No**
2	Does the author have symptoms of COVID-19?	**Yes**
3	Is a member of the author's household or family?	**Yes**
4	Does a member of the author's household or family have COVID-19?	**No**
5	Does a member of the author's household or family have symptoms of COVID-19?	**No**

Why? –The author talks about having infection in October and the date of post is from November, which is one month away. Therefore Author having covid is given label Yes. Furthermore, it says 'Wife had before me', so we can consider the time difference between the post and wife's infection may be more than 1 month (greater than our time of interest), that's why, Family Covid is given label No.

EXAMPLE 9:
Date: 2020-11-03
Text: ***"I had all these symptoms in September. Put it down to man flu. The fatigue was the worse. Did I have Covid. I don't know. Wife had before me."***
Is the text about...

1	Does the author have COVID-19?	**No**
2	Does the author have symptoms of COVID-19?	**No**
3	Is a member of the author's household or family?	**Yes**
4	Does a member of the author's household or family have COVID-19?	**No**
5	Does a member of the author's household or family have symptoms of COVID-19?	**No**

Why? – The author talks about having infection in September and the date of post is from November, which is two months away. Therefore, Author having covid is given label No.

EXAMPLE 10:
Date: 2021-04-01
Text: *"Well, me and my husband had COVID in March. We have a 7yo son. I would have liked to go to my family in Leeds (I am in Brighton) to help with childcare. We didn't. It genuinely irritates me."*
Is the text about...

1	Does the author have COVID-19?	**Yes**
2	Does the author have symptoms of COVID-19?	**No**
3	Is a member of the author's household or family?	**Yes**
4	Does a member of the author's household or family have COVID-19?	**Yes**
5	Does a member of the author's household or family have symptoms of COVID-19?	**No**

Why? – The author talks about having infection in March and the date of post is from April, which is one month away. Therefore, Author and Family having covid are given labels of Yes.

EXAMPLE 11:
Date: 2021-11-11
Text: *"My girlfriend has had #LongCovid symptoms now for 5 months after we both had Covid."*
Is the text about...

1	Does the author have COVID-19?	**No**
2	Does the author have symptoms of COVID-19?	**No**
3	Is a member of the author's household or family?	**Yes**
4	Does a member of the author's household or family have COVID-19?	**No**
5	Does a member of the author's household or family have symptoms of COVID-19?	**No**

Why? – The author talks about having covid 5 months ago which is outside the 1 month period of interest. This is why we label it as NO.

EXAMPLE 12:
Date: 2021-07-08
Text: *"I had flu last Christmas and my husband didn't. I'm also sure I had Covid in March and my husband didn't."*
Is the text about...

1	Does the author have COVID-19?	**No**
2	Does the author have symptoms of COVID-19?	**No**
3	Is a member of the author's household or family?	**Yes**
4	Does a member of the author's household or family have COVID-19?	**No**
5	Does a member of the author's household or family have symptoms of COVID-19?	**No**

Why? – The time difference between last Christmas and July is approx 7 months, thus this is given a label NO.

Supplementary References

[1] Jacob Devlin, Ming-Wei Chang, Kenton Lee, and Kristina Toutanova. Bert: Pre-training of deep bidirectional transformers for language understanding. *arXiv preprint arXiv:1810.04805*, 2018.

[2] Anders Giovanni Møller, Rob van der Goot, and Barbara Plank. NLP north at WNUT-2020 task 2: Pre-training versus ensembling for detection of informative COVID-19 English tweets. *Proceedings of the Sixth Workshop on Noisy User-generated Text (W-NUT 2020)*, pages 331–336, November 2020.

[3] Amil Merchant, Elahe Rahimtoroghi, Ellie Pavlick, and Ian Tenney. What happens to BERT embeddings during fine-tuning? *Proceedings of the Third BlackboxNLP Workshop on Analyzing and Interpreting Neural Networks for NLP*, pages 33–44, November 2020.

[4] Martin Müller, Marcel Salathé, and Per E Kummervold. Covid-twitter-bert: A natural language processing model to analyse COVID-19 content on Twitter. *Frontiers in Artificial Intelligence*, 6:1023281, 2023.
