# Supplementary material for: Estimating the household secondary attack rate and serial interval of COVID-19 using social media
Source: NPJ Digit Med. 2024 Jul 20;7:194. doi: 10.1038/s41746-024-01160-2 (PMC11271293; doi:10.1038/s41746-024-01160-2)
Supplement: Supplementary file 2 — Reporting Summary [file 41746_2024_1160_MOESM2_ESM.pdf]

nature portfolio

Corresponding author(s): Ingemar J. Cox

Last updated by author(s): Mar 15, 2024

Reporting Summary

Nature Portfolio wishes to improve the reproducibility of the work that we publish. This form provides structure for consistency and transparency in reporting. For further information on Nature Portfolio policies, see our Editorial Policies and the Editorial Policy Checklist.

Statistics

For all statistical analyses, confirm that the following items are present in the figure legend, table legend, main text, or Methods section.

n/a	Confirmed	
☐	☒	The exact sample size (n) for each experimental group/condition, given as a discrete number and unit of measurement
☐	☒	A statement on whether measurements were taken from distinct samples or whether the same sample was measured repeatedly
☒	☐	The statistical test(s) used AND whether they are one- or two-sided<br>*Only common tests should be described solely by name; describe more complex techniques in the Methods section.*
☐	☒	A description of all covariates tested
☐	☒	A description of any assumptions or corrections, such as tests of normality and adjustment for multiple comparisons
☐	☒	A full description of the statistical parameters including central tendency (e.g. means) or other basic estimates (e.g. regression coefficient) AND variation (e.g. standard deviation) or associated estimates of uncertainty (e.g. confidence intervals)
☒	☐	For null hypothesis testing, the test statistic (e.g. F, t, r) with confidence intervals, effect sizes, degrees of freedom and P value noted<br>*Give P values as exact values whenever suitable.*
☒	☐	For Bayesian analysis, information on the choice of priors and Markov chain Monte Carlo settings
☒	☐	For hierarchical and complex designs, identification of the appropriate level for tests and full reporting of outcomes
☐	☒	Estimates of effect sizes (e.g. Cohen's d, Pearson's r), indicating how they were calculated

Our web collection on statistics for biologists contains articles on many of the points above.

Software and code

Policy information about availability of computer code

Data collection	Online Twitter (now X) posts were collected using Python from an Application Interface (API) developed by Twitter and known as Twitter API V2 Academic Access. The software to download to data is included in our code availability repository.
Data analysis	We have used Python and associated packages for data analysis. The software to replicate the analysis is included in our code availability repository.

For manuscripts utilizing custom algorithms or software that are central to the research but not yet described in published literature, software must be made available to editors and reviewers. We strongly encourage code deposition in a community repository (e.g. GitHub). See the Nature Portfolio guidelines for submitting code & software for further information.

Data

Policy information about availability of data

All manuscripts must include a data availability statement. This statement should provide the following information, where applicable:

- Accession codes, unique identifiers, or web links for publicly available datasets
- A description of any restrictions on data availability
- For clinical datasets or third party data, please ensure that the statement adheres to our policy

Twitter only permits up to a maximum of 1,500,000 Tweet IDs to be distributed. Due to this limitation, we only provide the Tweet ID's of each of the tweets that are

positively classified by the three classifiers. We also provide the labelled data used to train the three classifiers. This is a total of 460,979 ID's. Data is available at https://figshare.com/s/375a15bbcca69af95822.

Research involving human participants, their data, or biological material

Policy information about studies with human participants or human data. See also policy information about sex, gender (identity/presentation), and sexual orientation and race, ethnicity and racism.

Reporting on sex and gender	NA
Reporting on race, ethnicity, or other socially relevant groupings	NA
Population characteristics	The study population comprises Twitter users residing in the United Kingdom who have posted at least one tweet mentioning either themselves or a household member contracting COVID-19 during the period spanning from January 1, 2020, to February 2022. As of 2021, Twitter demographics indicated that 58.5% of users identified as male, while 41.5% identified as female. Among age groups, individuals aged 25-34 comprised the largest proportion at 38.5%, whereas those aged 13-17 represented the smallest segment at 6.6% of UK Twitter users. However, it's important to note that any sample obtained may not necessarily reflect these population statistics exactly.
Recruitment	We did not engage in direct recruitment; rather, the study population was identified based on their Twitter activity. Individuals residing in the United Kingdom who shared posts about themselves or their household member contracting COVID-19 between January 1, 2020, and February 2022 were included in the study. There may be reporting bias with such participant selection, such that individuals with mild or asymptomatic cases may be less inclined to share their experiences online, leading to an underrepresentation of these cases.
Ethics oversight	This research has been conducted in accordance with ethical standards and principles. Approval for the study protocol, including the collection, analysis, and publication of data, was obtained from the UCL Research Ethics Committee (REC), (i) UCL REC 16621/003 ``Estimating the secondary attack rate and serial interval of COVID-19 using Twitter" and (ii) the UCL Computer Science REC /CSREC/R/30 ``Estimating the secondary attack rate and serial interval of COVID-19 using X, formerly known as Twitter".

Note that full information on the approval of the study protocol must also be provided in the manuscript.

Field-specific reporting

Please select the one below that is the best fit for your research. If you are not sure, read the appropriate sections before making your selection.

☐ Life sciences ☒ Behavioural & social sciences ☐ Ecological, evolutionary & environmental sciences

For a reference copy of the document with all sections, see nature.com/documents/nr-reporting-summary-flat.pdf

Behavioural & social sciences study design

All studies must disclose on these points even when the disclosure is negative.

Study description	Estimating the household secondary attack rate and serial interval of COVID-19 using social media data from Twitter (now called X)
Research sample	The analysis relies on a sample of Twitter posts originating from users in the United Kingdom. The selection process ensured that the sampled users had posted at least one tweet related to a COVID-19 infection between January 2020 and February 2022. The Twitter API retrieves the latest 3200 tweets for any given Twitter user.
Sampling strategy	At the outset, our sampling method focused on keywords and key phrases, collecting only Twitter posts that had the specified key phrases. Subsequently, users posting these relevant tweets were further sampled based on location, specifically targeting those located in the UK at the time of data collection. Utilizing the Twitter API, we gathered the most recent 3200 tweets for each selected user. To refine our dataset, users without tweets related to household members were subsequently excluded from the analysis.
Data collection	The data collection was performed automatically by custom python code using the Twitter Academic API.
Timing	January 1, 2020 to February 28, 2022
Data exclusions	After obtaining Twitter posts from the designated users, we implemented two exclusion criteria. Firstly, users lacking the original tweet for which they were initially chosen were removed. Secondly, individuals without tweets pertaining to household members were excluded from the analysis. This step was taken to mitigate potential biases in the dataset.
Non-participation	NA
Randomization	NA

Reporting for specific materials, systems and methods

We require information from authors about some types of materials, experimental systems and methods used in many studies. Here, indicate whether each material, system or method listed is relevant to your study. If you are not sure if a list item applies to your research, read the appropriate section before selecting a response.

Materials & experimental systems

n/a	Involved in the study
☒	☐ Antibodies
☒	☐ Eukaryotic cell lines
☒	☐ Palaeontology and archaeology
☒	☐ Animals and other organisms
☒	☐ Clinical data
☒	☐ Dual use research of concern
☒	☐ Plants

Methods

n/a	Involved in the study
☒	☐ ChIP-seq
☒	☐ Flow cytometry
☒	☐ MRI-based neuroimaging

Plants

Seed stocks	NA
Novel plant genotypes	NA
Authentication	NA
